# Generalized enrichment analysis improves the detection of adverse drug events from the biomedical literature

**DOI:** 10.1186/s12859-016-1080-z

**Published:** 2016-06-23

**Authors:** Rainer Winnenburg, Nigam H. Shah

**Affiliations:** Stanford Center for Biomedical Informatics Research, 1265 Welch Road, MSOB, Stanford, CA 94305 USA

**Keywords:** Enrichment analysis, Postmarketing pharmacovigilance, Information retrieval, MeSH indexing

## Abstract

**Background:**

Identification of associations between marketed drugs and adverse events from the biomedical literature assists drug safety monitoring efforts. Assessing the significance of such literature-derived associations and determining the granularity at which they should be captured remains a challenge. Here, we assess how defining a selection of adverse event terms from MeSH, based on information content, can improve the detection of adverse events for drugs and drug classes.

**Results:**

We analyze a set of 105,354 candidate drug adverse event pairs extracted from article indexes in MEDLINE. First, we harmonize extracted adverse event terms by aggregating them into higher-level MeSH terms based on the terms’ information content. Then, we determine statistical enrichment of adverse events associated with drug and drug classes using a conditional hypergeometric test that adjusts for dependencies among associated terms. We compare our results with methods based on disproportionality analysis (proportional reporting ratio, PRR) and quantify the improvement in signal detection with our generalized enrichment analysis (GEA) approach using a gold standard of drug-adverse event associations spanning 174 drugs and four events. For single drugs, the best GEA method (Precision: .92/Recall: .71/F1-measure: .80) outperforms the best PRR based method (.69/.69/.69) on all four adverse event outcomes in our gold standard. For drug classes, our GEA performs similarly (.85/.69/.74) when increasing the level of abstraction for adverse event terms. Finally, on examining the 1609 individual drugs in our MEDLINE set, which map to chemical substances in ATC, we find signals for 1379 drugs (10,122 unique adverse event associations) on applying GEA with *p* < 0.005.

**Conclusions:**

We present an approach based on generalized enrichment analysis that can be used to detect associations between drugs, drug classes and adverse events at a given level of granularity, at the same time correcting for known dependencies among events. Our study demonstrates the use of GEA, and the importance of choosing appropriate abstraction levels to complement current drug safety methods. We provide an R package for exploration of alternative abstraction levels of adverse event terms based on information content.

**Electronic supplementary material:**

The online version of this article (doi:10.1186/s12859-016-1080-z) contains supplementary material, which is available to authorized users.

## Background

### Motivation and significance

In 2000 the annual cost of drug-related morbidity and mortality was estimated to be $177.4 billion and rising [1]. In 2012 alone (the most recent year for which this data is available from the Agency for Healthcare Research and Quality) there were more than 1.9 million emergency department visits in the United States for adverse drug reactions [2]. Adverse drug events (ADE) are often missed in pre-market approval clinical trials due to small patient cohort sizes, exclusion of high-risk populations, and short follow-up times [3, 4]. The latter is of concern, since the risk for some adverse events increases with the time of exposure and the cumulative dosage of the drug [5]. Furthermore, adverse events might be caused by several drugs interacting with each other when administered concomitantly, and it is infeasible to systematically test a given drug for adverse interactions with each of the approved and experimental drugs via in vitro and in vivo methods [6].

Traditionally, drug safety monitoring relies on data from spontaneous reporting systems (SRS), such as the US Food and Drug Administration (FDA) Adverse Event Reporting System (FAERS) [7], which contain reports of suspected ADEs submitted by healthcare providers, manufactures, and patients. The reports in SRS are analyzed for drug-adverse event associations (also called safety signals) via statistical methods based on disproportionality measures, such as the reporting odds ratio (ROR) and the proportional reporting ratio (PRR), which quantify the magnitude of difference between observed and expected rates of particular drug-adverse event pairs [8, 9]. The FDA screens the FAERS database for the presence of an unexpectedly high number of reports of a given adverse event for a drug product using the empirical Bayes multi-item gamma Poisson shrinker (MGPS) data mining protocol, which includes stratification steps to minimize false positive signals [10]. Montastruc et al. list benefits and strengths of the disproportionality analysis for identification of ADEs in a pharmacovigilance database [11]. However, there are known limitations of such systems, such as the varying quality of reports and underreporting [12, 13].

As a result, increasingly there are efforts to use other data sources, such as electronic health records (EHRs), for detecting potential new ADEs [14] and complement signals seen in FAERS [15, 16]. Researchers have also used billing and claims data for active drug safety surveillance [17, 18] as well as turned to social media [19], clinical trial repositories [20], and literature mining for drug safety [21, 22]. In addition, there is work on aggregating ADEs at the level of drug classes [21, 23], learning drug interactions [24], and reasoning over literature to discover drug-drug interactions based on properties of drug metabolism [25]. In previous work using the literature for detecting drug safety signals [21], in which the proportional reporting ratio (PRR) was used to detect adverse drug class effects, one key problem was the choice of an appropriate level (in the hierarchy of a terminology) at which adverse event terms associated with drugs should be grouped into. This problem, of the lack of an appropriate, consistent, hierarchical abstraction level of adverse events, has also been noted before [8, 22].

Given the similar nature of disproportionality analysis and enrichment analysis (EA), we explore solutions to this abstraction level problem based on recent developments in EA. EA is commonly used to determine whether the Gene Ontology (GO) terms [26, 27] representing specific biological processes, molecular functions, or cellular components are over- or under-represented in the annotations of the genes deemed significantly altered in an experiment [28]. EA examines for disproportionality among the expected and observed counts of genes with a specific function or activity using a hypergeometric distribution model. While the GO has been the principal focus for EA, it is possible to perform EA using disease ontologies—such as SNOMED CT (Systematized Nomenclature of Medicine—Clinical Terms) [29]. For example, by annotating protein mutations with disease terms, Mort et al. identified a class of diseases—blood coagulation disorders—that are associated with a significant depletion in substitutions at O-linked glycosylation sites [30]. We can also apply the EA methodology to other datasets—such as patient cohorts. We refer to EA applied to non-traditional use cases as generalized enrichment analysis (GEA) [31, 32]. For example, GEA can detect specific co-morbidities that have an increased incidence in rheumatoid arthritis patients—a topic of recent discussion in the literature and considered essential to provide high quality care [33–35].

We believe that GEA can also be used to analyze drugs or a set of drugs, e.g., from a drug class, for associated adverse event terms. Counts of associated adverse event terms and single drugs can be gathered from the literature (e.g., based on MEDLINE indexing of drug related articles) and compared against expected counts based on background frequencies from a large reference set (e.g., all articles in the MEDLINE corpus). When using GEA, it is possible to address the issue of inconsistent hierarchies in terminologies via the use of *abstraction levels* [36]. An abstraction level is a subset of terms from a terminology that have similar specificity of meaning, and are independent of one another. Note that terms in a single abstraction level are not required to be at the same hierarchical level in a terminology—terms at a given abstraction “level” are just a set of terms that are statistically independent and have the same specificity.

In this paper, we develop an approach based on generalized enrichment analysis to examine associations between drugs and adverse events at multiple levels of granularity and simultaneously correct for known dependencies among predicted events. Our main contribution is the transfer of an established methodology in functional genomics to pharmacovigilance to address the problem of picking the right granularity of terms and the implementation of this approach as an R-package. As part of this effort, we have developed a compiled reference set of MeSH term frequencies from MEDLINE that can be used for EA studies in other use cases. We also provide a set of reusable adverse event terms by grouping the corresponding MeSH descriptors onto abstraction levels with uniform information content.

We validate our work using an established gold standard for drug safety signaling and comparing our results to an established drug safety signal detection method from the literature. We demonstrate improvement in the detection of associations between drugs and adverse events from the biomedical literature, by using the appropriate level of specificity for the adverse event terms. We highlight the potential and limitations of our approach on an example of a drug that is currently being investigated for its potential association with bladder cancer. As the published literature increasingly becomes a complementary source for post-marketing surveillance, our findings should be of interest to the curators of ADE repositories and drug safety professionals.

## Methods

**Fig. 1:**
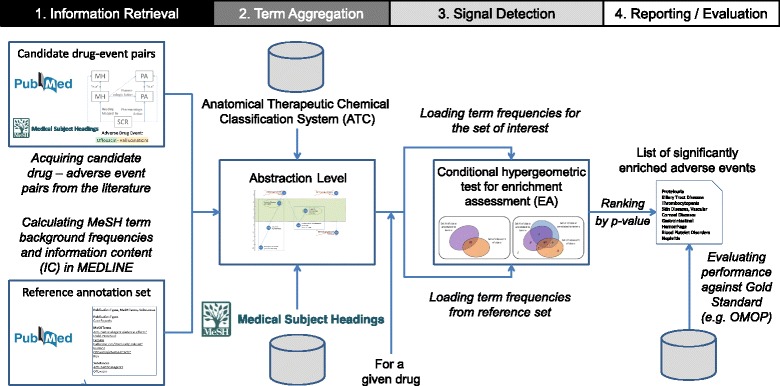
Overview on our approach for using generalized enrichment analysis for detecting adverse drug events

Our approach for using generalized enrichment analysis (GEA) for detecting adverse drug events (ADE) is depicted in Fig. 1 and can be summarized as follows: First, we acquire candidate associations between adverse events and drugs from MEDLINE articles from previous work in the form of MeSH term pairs. We precompute term frequencies and information content (IC) for all MeSH descriptors using the 2015 MEDLINE®/PubMed® baseline corpus (1). We then determine the level of granularity for the disease terms, using the precomputed information content (IC) for all MeSH descriptors in the entire MEDLINE 2015 reference set. We establish several abstraction levels by aggregating adverse events into higher-level MeSH disease terms based on their IC (2). For each drug, using the corresponding MEDLINE abstracts, we perform GEA to identify adverse events mentioned at an unexpectedly higher rate as compared to the reference set, which provides the expected frequency of the event being mentioned in the MeSH annotations of a set of MEDLINE abstracts. We perform a conditional hypergeometric test to calculate p-values that are corrected for known co-occurrence relationships between adverse event terms at several abstraction levels (3). Finally, we quantify the improvement in detecting true signals using a gold standard and comparing with standard methods (4).

### Acquiring candidate drug – adverse events from the literature

Efforts to extract ADEs from textual sources such as EHRs, social media, and the literature can rely on either natural language processing (NLP) (e.g., [37]) or simpler entity recognition of drug and disease terms, followed by supervised learning (for example, to distinguish the adverse events of a drug from the drug’s indications [38]). In addition, there are approaches that extract ADEs from articles in MEDLINE based on the manually assigned Medical Subject Headings (MeSH) terms for MEDLINE indexing (e.g., [22, 39, 40]).

For the first step in this study, the acquisition of candidate ADE pairs, we use the set of candidate ADE pairs between individual drugs and adverse events extracted from MEDLINE index terms from [40]. The goal is to prevent testing millions of associations that would need to be tested if we examined associations among all drugs (~5000) and all events (~10,000). This dataset consists of candidate ADE pairs extracted from MeSH term indexes of all 360 k articles in MEDLINE that are indexed with certain combinations of MeSH terms and qualifiers. The creation of this dataset (i.e., the MEDLINE query and subsequent recognition and filtering steps) is described in detail in [40], but in essence, from all index terms for a given article, ADE pairs were generated from combinations of a MeSH descriptor (or supplementary concept) and a qualifier, where one represents a drug involved in an ADE (e.g., *ofloxacin/adverse effects*) and the other represents a manifestation of an ADE (e.g., *tendinopathy/chemically induced*). Of note, in this example, *ofloxacin* and *tendinopathy* are MeSH descriptors, *adverse effects* and *chemically induced* are qualifiers that denote the context of the respective descriptor, and the resulting ADE pair is (*ofloxacin*, *tendinopathy*). All pairs of such qualified drugs and events that co-occur in the index of a given article in the set of 360 k articles are used as candidate ADE pairs.

In our study, we extract drugs and events into two separate files with the article ID as provenance information so they can be separately filtered and mapped to higher-level terms or classes, and later re-consolidated into ADE pairs (e.g., *ofloxacin* – *tendinopathy or ofloxacin – muscular diseases*) based on shared article IDs. Overall, we examine 105,354 unique ADE pairs (377,974 instances) from the full data set between 3057 event terms and 1609 drugs that are deemed clinically relevant according to RxNorm and can be mapped to ATC ingredients as described in [40]. RxNorm is a standardized nomenclature for medications produced and maintained by the U.S. National Library of Medicine (NLM) [41]. ATC is the Anatomical Therapeutic Chemical classification system of active ingredients of drugs developed by the World Health Organization Collaborating Centre for Drug Statistics Methodology (WHOCC) [42].

#### Mapping drugs to drug classes

We map all drugs from the drug-manifestation pairs extracted from MEDLINE to our target terminology, ATC. As described in [21], we map drugs through RxNorm ingredients, which are linked in RxNorm to ATC and MeSH identifiers. For example, the RxNorm drug *rosuvastatin* (RxCUI: 301542) is linked to both the MeSH drug *rosuvastatin* (MeSH ID: C422923) and the 5th-level ATC drug *rosuvastatin* (ATC code: C10AA07).

A given drug can be represented multiple times in ATC. Typically, topical drugs and systemic drugs have different ATC codes for the same active moiety. For example, the anti-infective *ofloxacin* has two codes in ATC, depending on whether it is classified as an antibacterial drug for systemic use (J01MA01) or as an ophthalmological drug (S01AE01). However, we consider unique ingredients when we associate drugs with their ADEs. We only use the codes to link drugs to their classes. For example, we would aggregate *ofloxacin* into the two *Fluoroquinolones* drug classes (J01MA and S01AE).

### Preparing a reference annotation set

For performing enrichment analysis, we need a large reference annotation set that provides us with expected MeSH term frequencies (also called background frequency). In addition, these frequency counts also allow us to calculate the information content of each term which is used to define a set of MeSH terms with similar specificity, referred to as an abstraction level. We also calculate pairwise co-frequencies of MeSH terms from the reference set as a basis for quantifying known dependencies among adverse event terms. For our work, the MeSH annotations for the entire 2015 MEDLINE serve as the reference annotation set. Of note, the set of interest and the reference set in our study are not mutually exclusive. The idea behind generalized enrichment analysis is that a small, specific set of interest is compared against a much larger, general reference set, which is why mutual exclusiveness is not a requirement.

#### Calculating term frequencies

We calculate term frequencies for all MeSH descriptors by counting the number of articles that are indexed with a given term in the MEDLINE 2015 baseline set^1^ divided by the total number of articles in the set. First, we calculate the sets of article IDs to which a given MeSH descriptor is assigned based on the file *MH_items.gz*. For each descriptor, we also assign the corresponding articles to all ancestor terms along the MeSH tree number hierarchy.^2^ For example, whenever an article is indexed with the term *Depressive Disorder* (tree number F03.600.300) we add the article ID also to the article sets of its ancestor terms, i.e., *Mood Disorders* (F03.600) and *Mental Disorders* (F03). Finally, we update the article counts for all MeSH descriptors according to the aggregated article sets.

#### Calculating Information Content (IC) scores

The information content of a given term *t*, *IC(t)*, is defined as shown in Eq. (1), where *p(t)* is the normalized probability distribution for the term with respect to all terms (calculated as shown in Eq. (2)).

1 $$ IC(t) = -{ \log}_2p(t) $$

2 $$ p(t) = \frac{\left|a(t)\right|}{\left|{\cup}_ia(i)\right|} $$

In practice, we calculate the IC for each MeSH term by taking –log2 of the number of articles annotated with this term and all its descendants, *a(t)*, divided by the total number of articles in the MEDLINE baseline reference annotation set, $\bigcup_ia(i)$. As a result, the IC quantifies the specificity of a term in the context of the large reference set, where terms annotating many articles, such as *Brain Diseases*, are expected to be general terms and are assigned a low IC. Terms annotating only a few articles, such as *Ischemic Attack*, *Transient*, are specific terms with a high IC.

#### Calculating term co-frequencies

Enrichment analysis and measures of “unexpectedness” assume that the probabilities with which two terms appear are independent of each other. However, that assumption is not always true. For example, mentions of Diabetes and Metformin are not independent because Metformin *treats* Diabetes. Statistical tests need to be cognizant of the degree to which two terms are expected to co-occur because of such dependencies. Terms that frequently co-occur in a large reference set are also expected to co-occur in the results of enrichment analyses by chance. This is especially true for hierarchically related terms (since annotations are also attributed to ancestor terms) but also non-hierarchical relations, e.g., common co-morbidities. One way to quantify such dependencies is by using the terms’ co-frequencies in a reference annotation set.

In this study, we calculate the pairwise co-frequency for a pair of MeSH terms by intersecting their sets of aggregated article IDs that are annotated with each term. The resulting co-frequency counts are stored in a co-frequency table. Theoretically this would have resulted in approx. 750 million co-frequencies for 27,455 MeSH descriptors. In practice, many terms never co-occur and the co-frequencies are undirected, so we only calculated the upper diagonal matrix and for only those terms that co-occur at least once. This way the total number of stored co-frequencies for our reference set is approx. 75 million term pairs.

### Aggregating adverse events into higher level disease classes

Adverse events can be expressed at different levels of granularity. In MEDLINE, the specificity of terms used for annotating a given article is subject to the findings in that article and to the availability of MeSH terms for a particular adverse event at annotation time. For analytical purposes, i.e., data mining on a large corpus of articles, it is advantageous to group individual specific terms into broader categories of related terms, because variation in the usage of highly specific terms will even out. Another advantage of abstracting from specific terms is the reduction of the total number of terms used for enrichment analysis (i.e., feature reduction) and minimization of hierarchical dependencies between these terms (ensuring feature independence). The same aggregation strategy is applied to both the terms from the set of interest (i.e., articles supporting ADEs for a given drug) and those in the reference set (i.e., all MEDLINE). We consider aggregation approaches based on the hierarchical levels in an ontology as well as those based on the information content.

#### Aggregating at a fixed hierarchical level in MeSH

The MeSH hierarchy has multiple levels. In a previous study [21], the 2nd level was selected as an appropriate abstraction level for adverse events. The level of a given MeSH term is reflected by its tree number. For example, we would aggregate the 3rd-level terms *Tendinopathy* (tree number C05.651.869) and *Rhabdomyolysis* (C05.651.807) to the 2nd-level descriptor *Muscular Diseases* (C05.651).

#### Aggregating using information content-based abstraction levels

An abstraction level is a subset of terms that have similar specificities and are ontologically independent of one another. Terms at a given abstraction level are not required to be at the same hierarchical level in the ontology hierarchy. For example *Tennis Elbow* and *Bone Diseases*, which are both terms at the 2nd level in the MeSH tree hierarchy, are of different specificity. Thus, instead of selecting terms at a specific hierarchical level we will use information theory to define abstraction levels.

Information content (IC) [43] provides a measure for characterizing the true distribution of information at various, perhaps uneven, levels of the ontology. We described how to compute the IC of MeSH terms based on their usage in a large reference corpus (MEDLINE) above. Ideally, only terms with one specific IC value should be selected to ensure independence. However, doing so would reduce the number of available terms drastically; therefore, in practice, terms within a certain range of IC are selected (Fig. 1) and the remaining dependencies between terms are corrected using the expected co-frequency information.

The appropriate range of IC scores will depend on the given use case and the ontologies used therein and has to be determined empirically. Typically, for diseases used in the context of patient cohort definitions or drug safety profiles, terms with IC < 3 tend to be too general and terms with IC > 10 tend to be too specific for enrichment analysis efforts. In this study, we tested several abstraction levels all with an upper limit < 10 and compared their impact on the results.

In the following, we will explain the term aggregation approach exemplarily, using an abstraction layer with a range from IC 4–7.5.

**Fig. 2:**
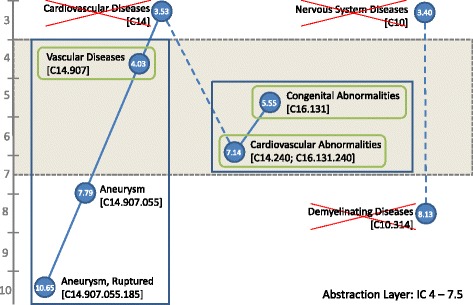
Defining an abstraction level based on information content (IC) of terms. Blue boxes denote terms that are considered under the given abstraction layer (here at IC range from 4.0 to 7.5); green circles denote terms that are used for representing these term aggregations, crossed out terms are ignored. Values in blue circles denote the information content (IC) of the term. Examples: Left box: *Aneurysm, Ruptured* and *Aneurysm* are aggregated into and represented by *Vascular Diseases*. Center box: *Cardiovascular Abnormalities* and *Congenital Abnormalities* are considered but *Cardiovascular Diseases* is ignored (too general). *Demyelinating Diseases* will not be considered under this abstraction layer because there is no ancestor term into which they could be aggregated. Tree numbers in square brackets indicate hierarchical relations between terms according to the MeSH tree hierarchy

Among all MeSH descriptor terms, we aggregate terms that have an IC higher than the upper limit of our target range (IC ≥ 7.5) to ancestor terms with IC within our target range (see Fig. 2). For example, we would aggregate both *Aneurysm, Ruptured* (IC 10.65) and *Aneurysm* (IC 7.79) into *Vascular Diseases* (IC 4.03). Under the given abstraction layer all three terms would be considered (left blue box in Fig. 2) but represented by *Vascular Diseases* (green circle). The term *Cardiovascular Abnormalities* (IC 7.14), which has two direct ancestor terms from two different branches in the MeSH C-tree, would be kept together with *Congenital Abnormalities (IC 5.55)* but not *Cardiovascular Diseases* (too general, IC 3.53). Both terms are considered (center blue box) and represented by themselves (green circles). Terms that cannot be aggregated into terms within the IC target range or that have an IC that is lower than the lower limit (e.g., IC < 4) are excluded. For example, neither the term *Demyelinating Diseases* would be considered (IC of 8.13 is too specific) nor its only direct ancestor term *Nervous System Diseases* (IC of 3.40 is too general).

### Detecting signals

Several signal detection methodologies for drug safety management exist [9], of which most are targeted at databases of spontaneous postmarketing adverse event reports, which include FDA FAERS in the US [7], the global WHO Vigibase [44], or EudraVigilance [45] in Europe. In signal detection, basic signal scores are based on disproportionality analysis, where the observed frequency of a drug – adverse event combination is compared with the expected frequency defined as a product of the individual frequencies of drug and adverse event in the corpus. The proportional reporting ratio (PRR) [46] is widely used in this context and is defined as

3 $$ PRR = \frac{a/\left(a+b\right)}{c/\left(c+d\right)} $$

where *a* is the number of reports that mention the drug and the adverse event, *b* the number of reports mentioning the drug without the adverse event, *c* the number of reports mentioning the adverse event without the drug, and *d* the number of reports neither mentioning the drug nor the adverse event.

To compensate for the over-sensitivity of disproportionality scores to small cell sizes (e.g., very rare events, drugs with only few reports), Bayesian methods have been introduced that make use of a prior distribution, representing existing knowledge with respect to the parameter of interest [9]. For example, the Bayesian confidence propagation neural network (BCPNN) has been developed by the WHO’s Uppsala monitoring centre (UMC) [47] and gamma poisson shrinker (GPS) and multi-item gamma poisson shrinker (MGPS) approaches are used by the FDA [10].

In this paper, we attempt signal detection using enrichment analysis, which is related to Bayesian methods because it also considers information on background term frequencies. We will evaluate the performance of our approach by using PRR as a baseline for signal detection on a common gold standard set of drug-adverse event associations.

#### Signal detection using GEA

R packages for enrichment analysis exist, notably two approaches which use MeSH as the term source. The *MeSH ORA framework* is a R/Bioconductor packages to support MeSH over-representation analysis [48]. *DOSE* is an R/Bioconductor package for disease ontology semantic and enrichment analysis [49]. Guided by these efforts, we developed an R package to compute the significance of associations between drugs (or drug classes) and adverse events from the set of extracted ADE candidates.

The main difference in our approach is the use of the correction applied to the p-value calculation based on known co-frequencies of terms. The current version of our package works with MeSH terms and uses MEDLINE 2015 baseline as the reference annotation set. The package can be extended to include additional domain specific ontologies and additional reference frequency values, such as those based on notes from EHRs [50].

#### Loading reference set with abstraction level

When a GEA object is instantiated with a specified abstraction level (e.g., IC 7–10), the corresponding abstraction layer of MeSH terms will be materialized: for all terms in the abstraction layer, aggregated term frequencies, corresponding IC scores, and term co-frequencies are loaded using values obtained from the MEDLINE 2015 baseline reference set as described earlier.

#### Loading term associations for the set of interest

For a given drug (or drug class), the *set of interest* constitutes all IDs of the articles in which the drug (or the drugs of a given drug class) are discussed in the context of some adverse event (step 1 in Fig. 1). These ADE associations are loaded in form of two tables containing the drug and event MeSH terms, respectively, which are associated through the corresponding MEDLINE article IDs. The terms in the *set of interest* are aggregated into higher-level disease terms from the specified abstraction level to make them comparable to the annotations from the *reference set*.

#### Adjusted hypergeometric test for enrichment assessment

The *conditionalHypergeometricTest* function is called with the *set of interest* (articles related to the drug and adverse events) from the previous step. The function returns a table of statistically enriched disease terms in the annotations of the articles in the *set of interest* corresponding to a given drug. The terms in the table are ranked by conditional *p*-values that are calculated as follows.

##### Step 1: Term-by-term enrichment analysis

**Fig. 3:**
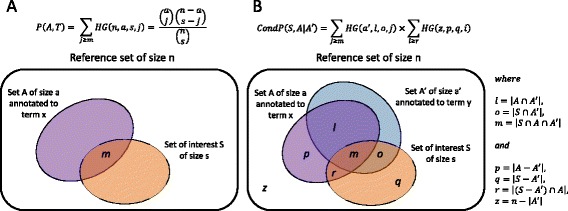
Differences between term-by-term enrichment analysis (**a**) and conditional enrichment analysis (**b**). See main text for details

In a first step (see Fig. 3a), p-values for each adverse event term *x* are calculated based on the overlap between the set of interest *S* and the reference set *A* performing hypergeometric tests, which calculate the probability of observing *m* or more articles annotated with term *x* in the set of interest *S* of size *s* given that *a* articles in the reference set of size *n* are annotated with term *x*. Terms that are statistically significantly enriched (e.g., *p*-value < 0.05) are deemed potentially associated with the drug in the set of interest.

##### Step 2: Correction for hierarchical relations between higher-level AE terms

The issue of dependencies among annotation terms from different branches of MeSH results from MeSH being a directed cyclic graph (DAG) rather than a tree and the 1:n assignment between MeSH descriptors and tree numbers. For example, the descriptor *Stevens-Johnson Syndrome* has six different tree numbers in MeSH, being classified as an *Immune System Disease*, *Stomatognathic Disease*, *Skin and Connective Tissue Disease* (3× at different levels within this branch), and *Chemically-Induced Disorder*. As a result, terms with multiple classifications, such as *Stevens - Johnson Syndrome,* will be aggregated into several higher-level terms at a given abstraction level.

Thus, in this second step (see Fig. 3b), adjusted *p*-values are calculated by determining pair-wise significance for each term given the other enriched terms in this set, by calculating the probability of observing *m* or more articles in the set of interest *S* of size *s* annotated with terms *x* and *y*, given that *o* items in the set of interest are also annotated with term *y*. The adjusted hypergeometric test takes into account the co-occurrences of terms in reference annotation sets to identify such dependency and will assign a larger *p*-value to the co-occurring term making it unsurprising.

For a given term, the final adjusted *p*-value is determined by the maximum of its entire pairwise conditional *p*-values. Only terms with an adjusted *p*-values < 0.05 (or any other selected threshold) are considered in the final list of enriched terms.

#### Performing baseline signal detection using PRR

We also use proportional reporting ratio (PRR) for computing statistical associations between drug (and drug class) – adverse event pairs. We compute PRR according to Eq. (3) using the frequencies that are calculated for each drug according to the contingency table shown in Table 1 based on the data extracted from the 360 k ADE related articles’ MeSH term indexes.

**Table 1:** 2 × 2 contingency table used for calculating associations between drugs and adverse events (AE) with proportional reporting ratio (PRR)

	With this AE	Without this AE
Articles mentioning the drug	a	b
Articles not mentioning the drug	c	d

We calculate PRR-based signals for all possible combinations of drugs and adverse events that co-occur in at least one of the 360 k relevant MEDLINE article. We apply the usual zero-cell correction by adding 0.5 to each count in 2 × 2 contingency tables with cells containing 0. For drug classes, we count articles mentioning any drug from this drug class (a and b) and articles mentioning any other drug (c and d). This approach is explained in more detail in [21].

### Evaluation

#### Gold standards

Only few gold standard reference sets for evaluating the accuracy of drug safety signal detection systems are publicly available. We evaluate the performance of signal detection using GEA at different levels of granularity against the drug safety reference set established by the Observational Medical Outcome Partnership (OMOP) [51]. This set contains 398 drug-outcome pairs, covering 181 drugs from several drug classes and four significant and actively monitored adverse event outcomes: *acute myocardial infarction*, *acute renal failure*, *acute liver injury*, and *upper gastrointestinal bleeding*. The OMOP gold standard is relatively independent from MEDLINE (the basis of our signal detection approach) because MEDLINE was not used to identify candidate positive controls but only to restrict the list of candidates that previously arose from product labeling and Tisdale review [51]. Other reference sets do exist, such as the also manually annotated EU-ADR corpus [52]. However, this reference set might be less applicable in the context of our study because the test cases were selected based on MEDLINE abstracts.

We compare signals retrieved from GEA with those retrieved from proportional reporting ratio (PRR). We apply signal detection methods at two different abstraction levels, for each of the four OMOP outcomes separately. We perform the comparison based on discrimination accuracy by calculating the areas under the receiver operator characteristic (ROC) curve (AUC). We perform this evaluation for drugs and drug classes separately.

#### Aligning MEDLINE data with the gold standard

We map the drug names in OMOP set to ingredients at the 5th level in ATC via RxNorm and the four outcomes to terms in the disease tree of MeSH. We were able to map all but 9 of the 183 unique drugs in the set. For the evaluation at the drug class level, we aggregated drugs into all its ATC4 classes. Because one drug may be mapped to multiple drug classes in ATC, the number of positive and negative associations might increase when mapping to the ATC4 drug class level. If drugs from the positive and negative control for a given outcome were aggregated into the same drug class, we removed that class from the negative class controls. For example, since *gatifloxacin* (J01MA16) is a negative control for *acute liver injury* but *ofloxacin* (J01MA01), *ciprofloxacin* (J01MA02), *norfloxacin* (J01MA06), *levofloxacin* (J01MA12), *trovafloxacin* (J01MA13), and *gemifloxacin* (J01MA15) are positive controls, we consider the drug class *Fluoroquinolones* (J01MA) as a positive control at the ATC4 class level. Table 2 shows the number of positive and negative controls that we consider for the four outcomes.

**Table 2:** Mapping of drugs and drug classes in the OMOP reference set to ATC

Aggregation	Drugs (ATC5)		Drug classes (ATC4)	
Outcome	Positive	Negative	Positive	Negative
Acute kidney injury	23	59	19	68
Acute liver injury	79	33	68	43
Acute myocardial infarction	33	61	18	72
GI bleed	24	62	19	73
Total	159	215	124	256

Positive and negative controls mapped to drugs and drug classes in ATC for the four adverse event outcomes in the OMOP reference set

The four adverse event outcomes in OMOP were manually mapped to MeSH descriptors at the relevant IC and tree hierarchy levels corresponding to the different abstraction levels used in this study. We chose the ranges for the three abstraction levels in such a way, that each outcome is represented by a different term (with different IC) at each level. Table 3 shows the MeSH terms (together with their descriptor IDs, level in the MeSH hierarchy, and their IC) that correspond to the four OMOP dataset outcomes at different levels of aggregation. For example, the two MeSH terms representing *acute kidney injury* in the literature, namely *acute kidney injury* and *kidney tubular necrosis, acute*, are represented by the MeSH descriptor *acute kidney injury* with an IC of 9.34 at the LOW IC abstraction level, *kidney diseases* with an IC of 5.73 at the MEDIUM IC abstraction level, and *urologic diseases* with an IC of 5.16 at the HIGH IC abstraction level as well as at the 2nd level in the MeSH hierarchy.

**Table 3:** Adverse drug event outcomes in OMOP mapped to MeSH terms at different abstraction levels

Aggregation	ORIGINAL	LOW (IC 7–10)	MEDIUM (IC 4.5–7)	HIGH (IC 1–5.5)	MeSH 2nd level
Acute kidney injury	Acute kidney injury, Kidney tubular necrosis, acute	Acute kidney injury D058186 (5th level) IC: 9.34	Kidney Diseases D007674 (3rd level) IC: 5.73	Urologic Diseases D014570 (2nd level) IC: 5.16	Urologic Diseases D014570 (2nd level) IC: 5.16
Acute Liver injury	Drug-induced liver injury, Drug-induced liver injury, chronic	Drug-Induced Liver Injury D056486 (3rd level) IC: 9.87	Liver Diseases D008107 (2nd level) IC: 5.64	Digestive System Diseases D004066 (1st level) IC: 4.01	Liver Diseases D008107 (2nd level) IC: 5.64
Acute Myocardial Infarction	Myocardial infarction, Anterior wall MI, Inferior wall MI, Myocardial stunning, Shock, cardiogenic	Myocardial infarction D009203 (4th level) IC: 7.22	Myocardial Ischemia D017202 (3rd level) IC: 5.94	Heart Diseases D006331 (2nd level) IC: 4.61	Heart Diseases D006331 (2nd level) IC: 4.61
GI bleed	GI Hemorrhage, Hematemesis, Melena, Peptic ulcer hemorrhage	Gastrointestinal Hemorrhage D006471 (3rd level) IC: 9.00	Gastrointestinal Diseases D005767 (2nd level) IC: 4.87	Digestive System Diseases D004066 (1st level) IC: 4.01	Gastrointestinal Diseases D005767 (2nd level) IC: 4.87

## Results

### Effect of using different abstraction levels on drug and adverse event counts

Table 4 summarizes the ADE data extracted from MEDLINE (in the ORIGINAL column) and the different “views” on these data resulting from different aggregation strategies. As can be seen in the ORIGINAL column, the original dataset contained 105,354 unique co-mentions of a drug and an adverse event, comprised of 3057 unique adverse event (AE) terms and 1609 unique drugs that map to 565 drug classes at the 4th level in ATC.

**Table 4:** Representation of ADE information from the literature at different abstraction levels

Aggregation	ORIGINAL (MeSH terms as extracted from MEDLINE)	LOW (IC 7–10)	MEDIUM (IC 4.5–7)	HIGH (IC 1–5.5)	MeSH 2nd level
# unique candidate ADE pairs	105354	87415	49696	41705	51226
# unique higher-level AE terms	3057	607	156	99	297
# unique AE terms covered	3057	2428	2760	2875	3039
# unique drugs with AE associations	1609	1590	1602	1608	1608
# unique drug classes (ATC 4) with AE associations	565	565	564	565	565

The number of unique higher-level AE terms into which the 3057 original AE terms group into, ranges from 607 terms using IC 7–10 to 99 using IC 1–5.5. Upon aggregation to the 2nd level in the MeSH tree-hierarchy, results in 297 terms. When aggregating into the 607 terms at the LOW abstraction level, 629 (20 %) of the 3057 original terms are not covered because the IC for these terms and all of their ancestor terms are outside the selected abstraction level. As expected, almost all original terms are captured when aggregating up to the fixed 2nd level in MeSH (only 18 1st level terms are ignored).

We also examined, how many drugs are affected by these ignored original adverse event terms across all abstraction layers (IC-based and the 2nd level tree hierarchy). Only few drugs are affected: from 1609 individual drugs and 565 corresponding drug classes, only 19 drugs lose all their adverse event associations at the LOW IC abstraction layer, 7 drugs at the MEDIUM IC abstraction level, and one drug at the HIGH IC and at the 2nd level MeSH tree abstraction level.

### Calibration of signal detection threshold using a gold standard

We evaluated the performance using the OMOP reference standard and by varying levels of granularity (abstraction levels) for the adverse event terms. In the following we describe the evaluation of signals for individual drugs. For the evaluation at the drug class level we refer to the Additional files.

**Fig. 4:**
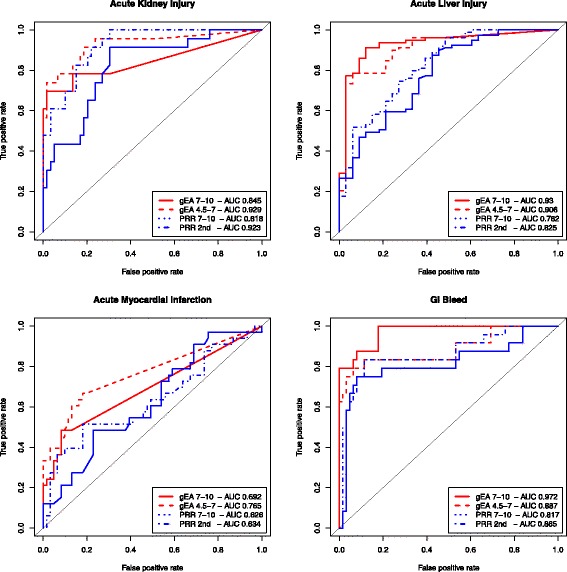
Performance of selected signal detection methods for single drugs on the OMOP reference set. Performance is measured for each of the four AE outcomes measured in terms of AUC summarizing all achievable combinations of true positive and false positive rates

We quantify performance of the signal detection using ROC curves summarizing all achievable combinations of true positive and false positive rates for each method for each of the four adverse event outcomes in the OMOP reference set (see Fig. 4 for single drugs and Additional file 1 for drug classes). We tested GEA (red lines) with two IC configurations (IC 7–10 (solid) and IC 4.5–7 (dashed)) and PRR (blue) with IC 7–10 (solid) and with fixed 2nd-level in MeSH hierarchy (dashed).

Both GEA and PRR methods perform best in detecting associations of drugs with *GI bleed* (AUCs from .817 to .972) and poorest on AMI (AUCs from .626 to .765). GEA has the biggest advantage over PRR for *acute liver injury* and *GI bleed*. Between the two PRR variants, PRR with fixed 2nd level terms outperforms PRR with IC 7–10. GEA with IC range of 4.5–7 performs better than the other methods for the AE outcomes *acute kidney injury* (AUC .929) and *acute MI* (AUC .765), although all methods perform poorly on the latter. PRR 2nd-level performs almost as well as the best GEA method on *acute kidney injury* (AUC .923 and .929, respectively) but worse on the other outcomes. GEA with IC range of 7–10 outperforms (AUC .930) the best PRR-based method (AUC .825) on *acute liver injury* and outperforms all other methods on *GI bleed* (AUC .972).

On grouping the drugs into drug classes (ATC4), the performance of the different approaches is similar for each of the four outcomes, with the one exception of PRR 2nd level on *acute myocardial infarction* (AUC drops to .592) as can be seen in Additional file 1.

The overall performance of enrichment (GEA) and the proportional reporting ratio (PRR) methods in terms of precision, recall, and F-measure at different p-value and PRR thresholds based the OMOP reference set is summarized in Table 5 (and Additional file 2 for drug class level results). Overall, GEA-based methods outperform PRR with a best F1-measure of .80 for GEA and .69 for PRR across the different configurations. Best recall is achieved using GEA at an abstraction level of IC 7–10 using a *p*-value < .1 (recall of .76) and best precision with the same method using a lower *p*-value (precision of .92 at *p* < .005).

**Table 5:** Overall performance measured on all single drugs and outcomes in OMOP gold standard

GEA	7–10			4.5–7			1–5.5			2nd level		
Threshold	0.1	0.05	0.005	0.1	0.05	0.005	0.1	0.05	0.005	0.1	0.05	0.005
TP	**121**	119	113	112	110	100	116	113	104	116	115	102
TN	182	188	**205**	191	195	**205**	159	164	178	186	193	198
FP	33	27	**10**	24	20	**10**	56	51	37	29	22	17
FN	**38**	40	46	47	49	59	43	46	55	43	44	57
Precision	0.79	0.82	**0.92**	0.82	0.85	0.91	0.67	0.69	0.74	0.80	0.84	0.86
Recall	**0.76**	0.75	0.71	0.70	0.69	0.63	0.73	0.71	0.65	0.73	0.72	0.64
F1-Measure	0.77	0.78	**0.80**	0.76	0.76	0.74	0.70	0.70	0.69	0.76	**0.78**	0.73
PRR	7–10			4.5–7			1–5.5			2nd level		
Threshold	1	1.5	5	1	1.5	5	1	1.5	5	1	1.5	5
TP	**112**	96	48	110	93	39	111	83	6	109	90	27
TN	126	144	197	150	172	208	152	176	**211**	166	184	**211**
FP	89	71	18	65	43	7	63	39	**4**	49	31	**4**
FN	**47**	63	111	49	66	120	48	76	153	50	69	132
Precision	0.56	0.57	0.73	0.63	0.68	0.85	0.64	0.68	0.60	0.69	0.74	**0.87**
Recall	**0.70**	0.60	0.30	0.69	0.58	0.25	**0.70**	0.52	0.04	0.69	0.57	0.17
F1-Measure	0.62	0.59	0.43	0.66	0.63	0.38	0.67	0.59	0.07	**0.69**	0.64	0.28

Performance with GEA (top) and PRR (bottom) using different abstraction levels and thresholds. Bold numbers indicate best performance throughout all configurations (individually for GEA and PRR-based configurations)

Assuming equal importance of false positives and false negatives, at the optimal F1-measure (.80 for single drugs and .79 for drug classes), GEA-based methods detect signals for single drugs with very high precision (.92) but are prone to generating false positives at the drug class level (precision of .71). At the drug class level, precision of > .8 can only be reached with accepting a moderate recall of .51.

PRR-based methods perform overall weaker than GEA, in particular with regard to recall. Best recall is achieved using abstraction levels of IC 7–10 and 1–5.5 for adverse event terms and PRR > 1 (recall of .70). Overall, PRR performs slightly better when using terms at the 2nd level in the MeSH tree hierarchy (best F1 .69). High precision comparable to that of the GEA methods is only achievable using PRR of >5 (precision of .87 at recall of .17).

### Detecting putative adverse drug event signals

We calculate the enrichment (GEA) and the proportional reporting ratio (PRR) of adverse events in the 105,354 candidate pairs between 1609 single drugs and 3057 AE terms that we had extracted from MEDLINE abstracts (see Table 4 for individual drugs and Additional file 3 for drug classes). We select the signaling thresholds and levels of aggregation of adverse event terms based on the best performances on the OMOP reference set (see Table 6). The numbers represent the distinct drug – adverse event pairs for which signals were found for a given method, abstraction layer, and threshold before p-value correction. The numbers in parentheses for the GEA-based methods represent pairs that passed the correction for known dependencies among enriched terms based on conditional *p*-values. On applying the optimal threshold (GEA at IC 7–10 with *p* < 0.005), we find signals for 10,122 unique adverse event associations (shown in bold in Table 6).

**Table 6:** Adverse event signals for drugs detected by GEA and PRR

*p*-value	GEA IC 7–10	GEA IC 4.5–6	GEA IC 1–5.5	PRR 2nd	PRR IC 7–10	PRR
<0.1	43702 (22073)	10585 (7634)	12875 (6157)	30547	61108	>1
<0.05	35953 (17140)	9249 (6840)	11453 (5356)	24834	52377	>1.5
<0.005	23059 (**10122**)	6867 (4931)	8594 (3623)	10810	26953	>5

Adverse event signals for drugs (ATC ingredient names) detected by GEA and PRR using different thresholds at different aggregation levels. The number in bold indicates the unique adverse event association signals found using the optimal configuration (GEA at IC 7–10 with *p* < 0.005) as determined by the overall performance assessment

### Test case: examining signal for pioglitazone and bladder cancer

We apply our approach to retrieve information for a possible ADE that is currently discussed in the drug safety community. *Pioglitazone* is indicated to lower blood glucose in adults with type 2 diabetes mellitus. After tumors had been observed in the urinary bladder of male rats in a premarketing two-year carcinogenicity study [53] (the tumorigenic potential of pioglitazone was also shown in a later animal study [54]), a 10-year observational study was started in 2003 to evaluate the potential risk of bladder cancer with pioglitazone use in humans. In a recently published research article [55], the authors conclude that *pioglitazone* was not associated with a statistically significant increased risk of bladder cancer but did not exclude the possibility of an increased risk. Although, this is in fact only the latest of several articles published in recent years focusing on the safety of *pioglitazone*, there is still insufficient data to determine whether pioglitazone is a tumor promoter for urinary bladder tumors [53].

Using our methodology to identify ADE signals from MEDLINE, we found the candidate association between *pioglitazone* and *urinary bladder neoplasms* in 28 articles. Although all articles were annotated with the MeSH descriptors in the context of adverse effects and chemically induced manifestations, a manual literature review revealed that four articles contained results from comparative cohort studies, another five contained results from retrospective/meta-analysis studies, one presented a case-report, whereas the rest comprised letters, comments, and review articles.

From the four comparative cohort studies, two concluded an statistically significant increased risk of bladder cancer [56, 57] whereas two did not [58, 59]. From the retrospective / meta-analysis studies, all [5, 60–62] but one [63] concluded an elevated risk. There is one study that found an association between pioglitazone use and bladder cancer based on reports from the U.S. Food and Drug Administration (FDA) Adverse Event Reporting System (FAERS) with a reporting odds ratio (ROR) of 4.30 [95 % CI 2.82–6.52] [61].

Based on the MeSH annotations from these 28 articles, all our signal detection methods found statistically significant association between *pioglitazone* and bladder tumors: GEA 7–10 to *Urinary Bladder Neoplasms* (*p*-value: 1.58e-08), GEA 4.5–7 to *Urogenital Neoplasms* (*p*-value: 5.05e-16), PRR with IC 7–10 to *Urinary Bladder Neoplasms* (PRR 93.40; 95 % CI 65.03–134.15), and PRR at 2nd level in MeSH tree hierarchy to *Urogenital Neoplasms* (PRR 38.59; 95 % CI 27.04– 55.07).

## Discussion

We present an approach based on generalized enrichment analysis (GEA) that can be used to signal associations between drugs, drug classes and events from the biomedical literature, at multiple levels of granularity. We validate our approach using an established gold standard for drug safety signaling and compare our results to an existing drug safety signal detection method. We demonstrate that adverse drug events can be observed at different levels of granularity, using drug class information in ATC for grouping drugs and information content of disease terms in MeSH for grouping adverse event terms.

While our results show a general advantage of GEA methods over PRR in detecting signals from the OMOP gold standard, perhaps resulting from the adjustments based on co-frequencies, the influence of abstraction on the performance is less obvious. The data from our evaluation suggests that increasing the level of aggregation to more than the outcome definitions in OMOP (e.g., IC 4.5–7) does not have a beneficial effect.

As an indirect contribution, we provide a meaningful aggregation of adverse event terms extracted from MEDLINE indexing by grouping the corresponding MeSH descriptors onto abstraction levels with uniform information content. The soundness of this approach is supported by the performance of GEA with IC 7–10 on the gold standard. However, for PRR we observed that selecting terms with IC 7–10 showed only minimal improvement over selecting terms at the second level in the MeSH hierarchy.

Although GEA performs better in the retrospective evaluation on the OMOP gold standard, it does not prove an advantage in a real world scenario of continuous real-time drug safety monitoring (prospective studies) [64]. However, our evaluation on the OMOP reference set suggests that PRR is indeed more prone to over-predicting signals (false positives), reflected by the fact that a precision similar to the one of the GEA methods could only be achieved at the cost of a high reduction of recall (GEA precision of .92 at a recall of .71 vs. PRR precision of .87 at a recall of .17, see Table 5).

### Varying performance across outcomes in the gold standard

We note that the performance varies significantly by event ranging from the best of AUC 0.97 for detecting associations with GI bleed to worst of AUC 0.69 for associations with Acute Myocardial Infarction (AMI). The four events are of different medical severity and hence are known to be affected by different reporting rates. For example, given that heart attacks are common due to other reasons, the reporting rates for such a common event tend to be lower (this effect has also been seen in prior work [65]).

Although underreporting is in general a well-known problem in spontaneous reporting systems, it also affects signal detection efforts using the biomedical literature as a source. We found that the coverage of ADEs in MEDLINE differs significantly across the four outcomes in OMOP (see Additional file 4). In particular for AMI, only 16 out of the 33 positive test cases (48 %) were found in our set of candidate drug – adverse events from the literature (at low abstraction level). For 17 out of 33 drugs, there was not a single article mentioning a relation between drug and AMI, and hence no signal could be detected. In contrast, for GI bleed all 24 (100 %) positive test cases could be found in at least one article, for acute liver injury 76 out of 79 (96 %), and for acute kidney injury still 18 out of 23 (78 %). Of note, while the presence of articles mentioning a relation does not automatically guarantee a strong signal, the absence of any article results in no signal.

Furthermore, MeSH does not provide a term specifically for AMI. The closest terms are *Myocardial Infarction* as well as *Anterior Wall MI* and *Inferior Wall MI*, which both contain the term *acute* in one of their synonyms. We further observed that drugs that are positively associated to AMI in OMOP tend to be annotated in the literature (if at all) with more high-level terms, such as *Heart Diseases* or *Cardiovascular Diseases*. Indeed, we find that - against the general trend - the number of true positive findings with GEA at a high abstraction level (IC 1–5.5) doubles from the number at low abstraction level (IC 7–10) (see Additional file 5). Of note, the true positive rate for MI is at the high abstraction level still much lower than for the other outcomes.

Based on this examination of our results and the data sources we used in our study, we hypothesize that the limited availability of appropriate terminology and MeSH indexing practices might influence signal detection for AMI more negatively than for the other three outcomes in OMOP.

### Impact of aggregation level

In this study we defined several abstraction levels, namely high abstraction as terms with IC 1–5.5, medium abstraction as terms with IC 4.5-7, and low abstraction as terms with IC 7–10. This process is use-case specific. Level and the range of the level have an impact on term selection. At abstraction level IC 7–10, 2424 (from 3057 in total) adverse event terms are represented as 607 higher-level terms, excluding 633 terms that cannot be represented at the chosen abstraction level. At abstraction level IC 4.5-7 the number of higher-level terms is reduced to 156 (covering 2760 terms), excluding fewer terms but also providing significantly less granularity.

Level and the range of the level have also an impact on the number of detected distinct drug events (see Table 6). It is generally expected to find more ADE pairs at IC 7–10 than, e.g., at IC 4.5–7, because for a given drug several specific but related terms might describe similar (or the same) adverse events while these terms might be aggregated into one single term at a higher abstraction level. However, as can be seen in Table 6, the correction for known dependencies (numbers in parentheses) levels this effect out to some extent as expected.

Grouping into such high-level disease terms might hinder accurate signal detection. For example, the adverse event *Gastrointestinal Hemorrhage* at level 7–10 would be represented as *Gastrointestinal Diseases* at level 4.5 -7, combining signals for gastrointestinal hemorrhage with those for peptic ulcer, gastrointestinal tuberculosis, and others.

Aggregating signals at the level of drug classes provides better recall according to our evaluation: at an F1-measure of .79 and .80 respectively, the best GEA method achieves a recall of .84 at a precision of .75 at the level of drug classes, in comparison to .71 at a precision of .92 at the level of individual drugs. For exploratory studies, applying GEA at higher abstraction level for both drugs and adverse events might be a feasible starting point, if followed by additional “drill-down” studies in which the adverse events are more accurately assessed at the level of individual drugs as suggested previously [21].

### Error analysis

In order to illustrate the kind of errors that may occur, we discuss the cases of false positive and negative predictions for the outcome *acute kidney diseases*.

#### False negatives

We miss signals with GEA at IC 7–10 for seven drugs from the 23 in the positive control set for *acute kidney diseases* at a threshold of *p* < 0.005.

The most common reason for missing a signal is the absence of support for an ADE in MEDLINE. For five of the seven drugs for which we cannot find a signal (*chlorothiazide, oxaprozin, etodolac, telmisartan, moexipril*), not a single candidate association could be extracted from MEDLINE. In the two other cases, there is only weak or indirect support, e.g., from animal studies. For example, there is a retrospective case–control study of the effects of long-term dosing with *meloxicam* on renal function in aged cats with degenerative joint disease [66], in which the effect is classified as *chronic renal insufficiency* rather than *acute kidney injury*. However, at a higher aggregation level both acute and chronic manifestations are classified as *kidney diseases*, and indeed, at IC 4.5–7 GEA signals a significant association for *meloxicam*. There is also a signal for *capreomycin* at level 4.5–7.

In contrast, using PRR > 1.0 and 2nd level aggregation in MeSH (detecting signals for the broader 2nd level term *Urologic diseases*), five drugs in the positive control do not have signals but two of the drugs that were missed by GEA methods do have signals (*telmisartan* 2.16 (1.01–4.60), *capreomycin* 2.81 (1.34–5.91)).

#### False positives

We find signals with GEA 7–10 for three drugs from the 59 cases in the negative control set for acute kidney diseases at a threshold of *p* < 0.005: *flutamide*, *orlistat*, and *retinol (vitamin A)*.

Often, such false positive signals are generated on the basis of a few case reports in MEDLINE with outdated or only indirect information. For example, for the drug *flutamide* the signal (p–value 0.0017) is based on three publications from the late 1990s and early 2000s. One case report describes a 54-year-old man with metastatic prostate cancer who developed nonoliguric acute renal failure during treatment with *flutamide*. The authors conclude that “although very rare, flutamide-induced acute renal failure should be considered” [67]. In other reports the role of the drug as the main source of the adverse event is unclear, e.g., because the major event is rather liver injury than renal failure [68] or the event is observed when the drug is administered together with other antineoplastic agents [69].

However, for one drug, *orlistat*, for which we find a signal with p-value 1.38e-03 using GEA 7–10, there are five recent articles in MEDLINE that support an association with *acute renal failure*, e.g., an analysis of 953 patients from 2011 [70]. Interestingly, also the product label for XENICAL, a branded drug of *orlistat*, mentions that “cases of oxalate nephrolithiasis and oxalate nephropathy with renal failure have been reported” [71]. This may indicate that such a signal, which is also found with PRR of 3.25 (95 % CI 2.01–5.25), might not be a false positive.

### Limitations

Given the size of the reference set and the performance variation across outcomes, generalizing the results is not straight forward. Arguably, we could have tested our methodology on a larger test set with more outcomes. However, as discussed earlier, other sets might be less appropriate in this context, such as the one provided by the EU-ADR project, which was created using the same source (i.e., published papers). Another limitation is that we only present a retrospective evaluation on established ADEs which does not assess the applicability of our methodology for prospective signal detection [64, 72]. However, with some modifications to the scripts provided in our GEA R package it should be possible to conduct a prospective study on time-indexed reference sets, such as the one published in [73].

While we used MeSH as the source of terms to represent adverse events, the Medical Dictionary for Regulatory Activities (MedDRA) offers an alternative terminology for the same purpose. However, using that would require a validated mapping between terms from MeSH to MedDRA, which is a problem that needs to be addressed of its own.

Another limitation is that some of the terms that GEA based methods find to be enriched for many drugs are rather unspecific, such as *Drug-Related Side Effects and Adverse Reactions*. This “side-effect” results from the calculation of enrichment in comparison to a large, general reference set (MEDLINE as a whole) instead of a more drug safety focused control set.

The example of *pioglitazone* suggests that our method is capable of detecting the articles relevant to a specific drug safety concern and detecting possible signals. Although this approach allows to determine the correct context of the drug (the adverse effect of the drug is subject of the article) and the disease (it is chemically induced) individually, it does not guarantee a causal relationship between the two, in particular when there are several drugs and/or events subject of the same article or the publications do not present original research such as comments and reviews. These limitations can lead to over-generating signals for some drugs, which could be mitigated by filtering for certain publication types (such as clinical trials or case reports) and applying natural language post-processing (NLP) on article abstracts (if available).

Finally, as stated by Montastruc et al. [11], it is important to note that disproportionality studies should be only considered as exploratory in a context of signal detection. Such literature mining does not replace but can complement existing pharmacovigilance efforts. It is reassuring that our approach could identify 75 % of drug – outcome pairs in the OMOP reference set at a precision of 82 % (GEA, IC 7–10, *p*-value <0.05) based on information published in MEDLINE alone.

## Conclusions

We provide a framework based on generalized enrichment analysis that can be used to detect associations between drugs, drug classes and adverse events at a given level of granularity, at the same time correcting for known dependencies among events. Our study demonstrates the use of GEA, and the importance of choosing appropriate abstraction levels to complement current drug safety methods. The soundness of this approach is supported by the high performance of GEA with IC 7–10 on the gold standard.

We provide an R package that allows the exploration of alternative abstraction levels for adverse event terms based on information content. We provide a pre-computed set of aggregations of adverse event terms extracted from MEDLINE indexing by grouping the corresponding MeSH descriptors onto abstraction levels with uniform information content.

### Ethics approval and consent to participate

Not applicable.

### Consent for publication

Not applicable.

### Availability of data and material

We made the R-package and all data sets used for this study available.

Project name: GEA.

Project home page: https://github.com/winnenbr/GEA.

Operating system: Platform independent.

Programming language: R (> = 3.1.0).

License: Apache License, Version 2.0.

## Additional files

Additional file 1: Performance of selected signal detection methods for drug classes on the OMOP reference set. Performance is measured for each of the four AE outcomes in terms of AUC using all achievable combinations of true positive and false positive rates after grouping the drugs into drug classes (ATC4). The performance of the different approaches is similar for each of the four outcomes, with the one exception of PRR 2nd level on acute myocardial infarction (AUC .612). Overall, both GEA and PRR methods perform best on GI bleed (AUCs from .897 to .935) and poorest on acute liver injury (AUCs from .812 to .853). Using PRR and AE terms aggregated to terms at IC 7–10 improved performance in detecting associations with drug classes (ATC4) only for the outcomes GI bleed and acute myocardial infarction. (PDF 9 kb) Additional file 2: Overall performance measured on all drug classes and outcomes in OMOP gold standard. Performance is measured for each of the four AE outcomes measured in terms of AUC summarizing all achievable combinations of true positive and false positive rates. (XLSX 11 kb) Additional file 3: Adverse event signals for drug classes detected by GEA and PRR. Adverse event signals for drug classes (ATC4 codes) detected by GEA and PRR using different thresholds at different aggregation levels. (XLSX 9 kb) Additional file 4: Coverage of ADEs in MEDLINE. Coverage of adverse drug events in MEDLINE differs significantly across the four outcomes in OMOP. In particular for “Acute myocardial infarction”, only 16 out of the 33 positive test cases (48 %) were found in our set of candidate drug – adverse event pairs from the literature (at low abstraction). For 17 out of 33 drugs, there was not a single article mentioning a relation between drug and acute MI, and hence no signal could be detected. (XLSX 9 kb) Additional file 5: True Positive Rates for the four OMOP outcomes at different abstraction levels. Drugs that are positively associated to “Acute Myocardial Infarction” in OMOP tend to be annotated in the literature (if at all) with more high-level terms, such as “Heart Diseases” or “Cardiovascular Diseases”. Thus, the number of true positive findings with GEA at a high abstraction level (IC 1–5.5) is double that from the number at low abstraction level (IC 7–10) for AMI. (PDF 4 kb)

## Abbreviations

ADE: adverse drug event
AE: adverse event
ATC: anatomical therapeutic chemical classification
AUC: area under the curve
EA: enrichment analysis
EHR: electronic health record
FAERS: FDA adverse event reporting system
FDA: US Food and Drug Administration
GEA: generalized enrichment analysis
GO: gene ontology
IC: information content
MedDRA: medical dictionary for regulatory activities
MeSH: medical subject heading
MGPS: multi-item gamma poisson shrinker
NLP: natural language processing
OMOP: observational medical outcomes partnership
PRR: proportional reporting ratio
ROC: receiver operating characteristic
ROR: reporting odds ratio
SNOMED CT: systematized nomenclature of medicine--clinical terms
SRS: spontaneous reporting system
WHO: World Health Organization

## Footnotes

1 Available at https://mbr.nlm.nih.gov/Downloads.shtml.

2 File mtrees2015.bin available at ftp://nlmpubs.nlm.nih.gov/online/mesh/2015/meshtrees/.
