# Supplementary figures and images for: Generalized enrichment analysis improves the detection of adverse drug events from the biomedical literature
Source: BMC Bioinformatics. 2016 Jun 23;17:250. doi: 10.1186/s12859-016-1080-z (PMC4918084; doi:10.1186/s12859-016-1080-z)

**Acute Kidney Injury**

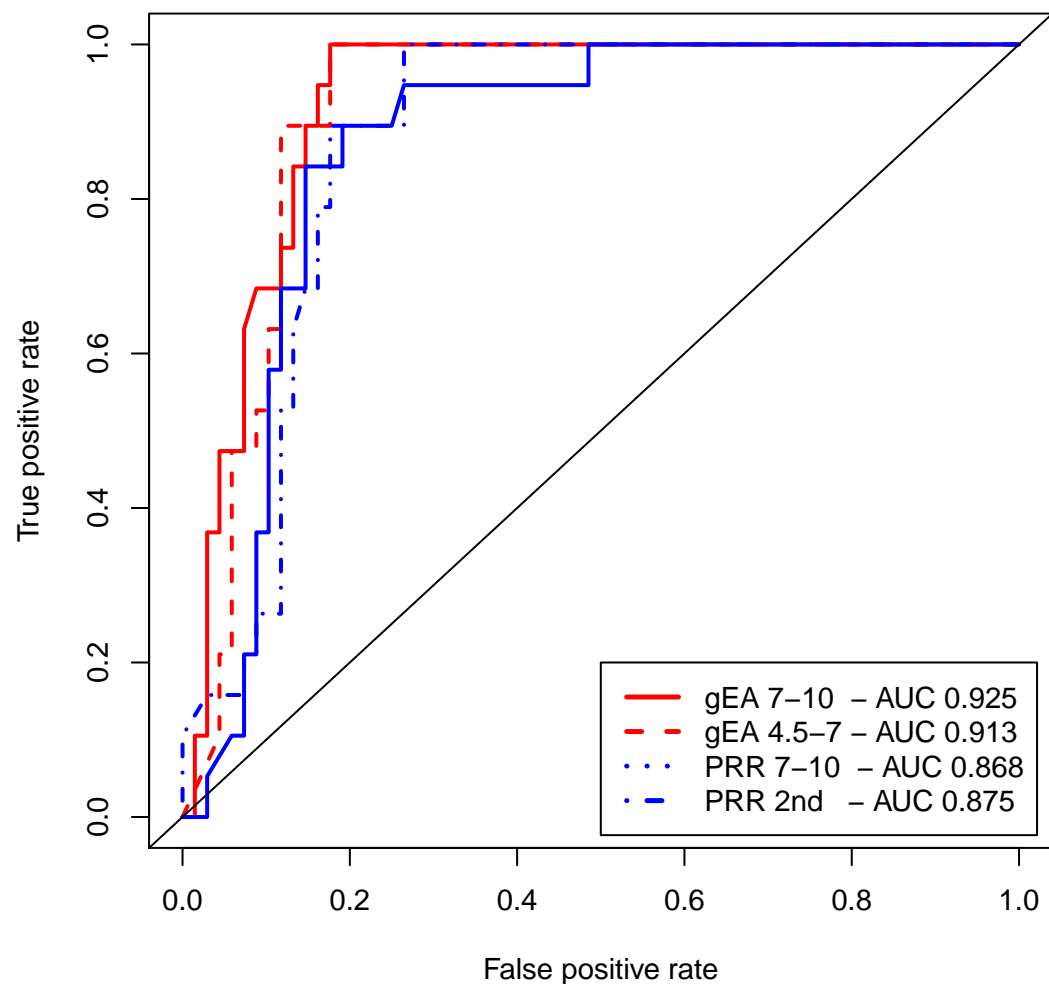

**Acute Liver Injury**

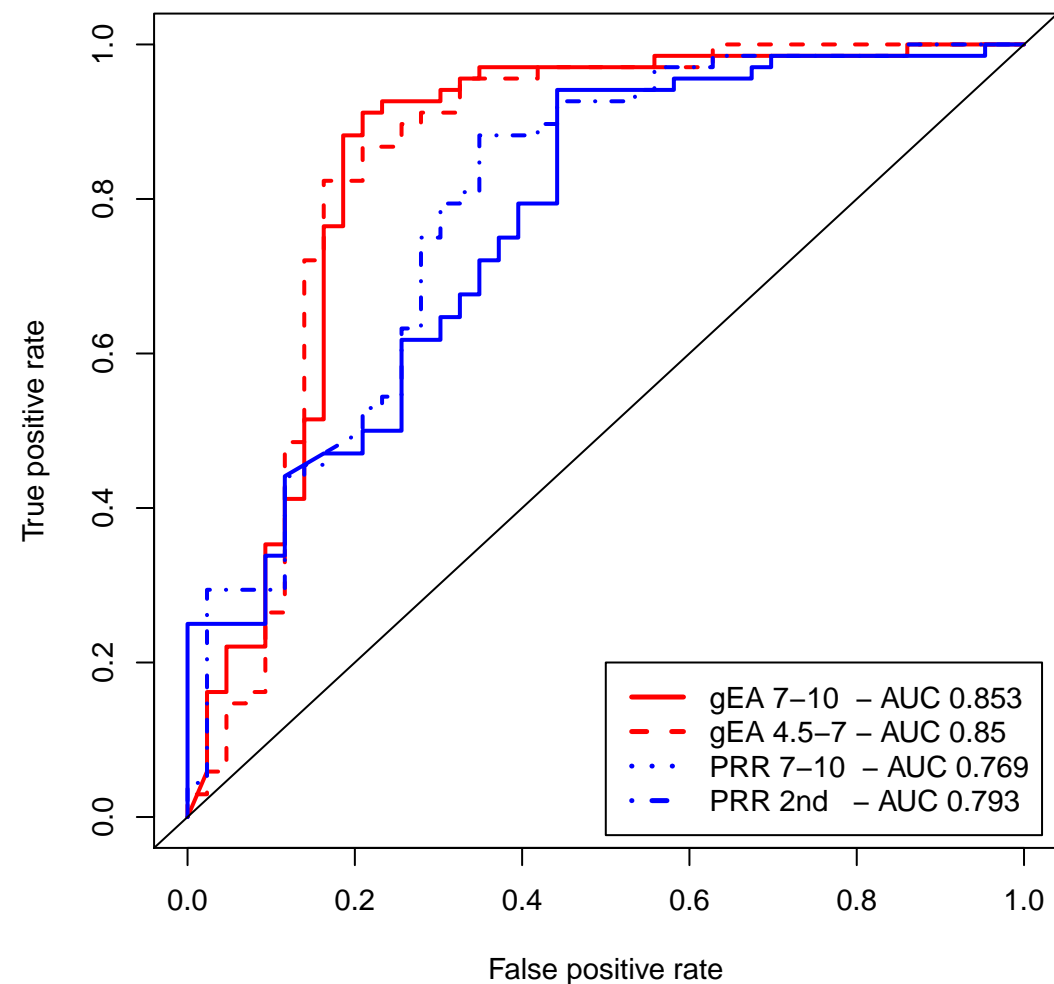

**Acute Myocardial Infarction**

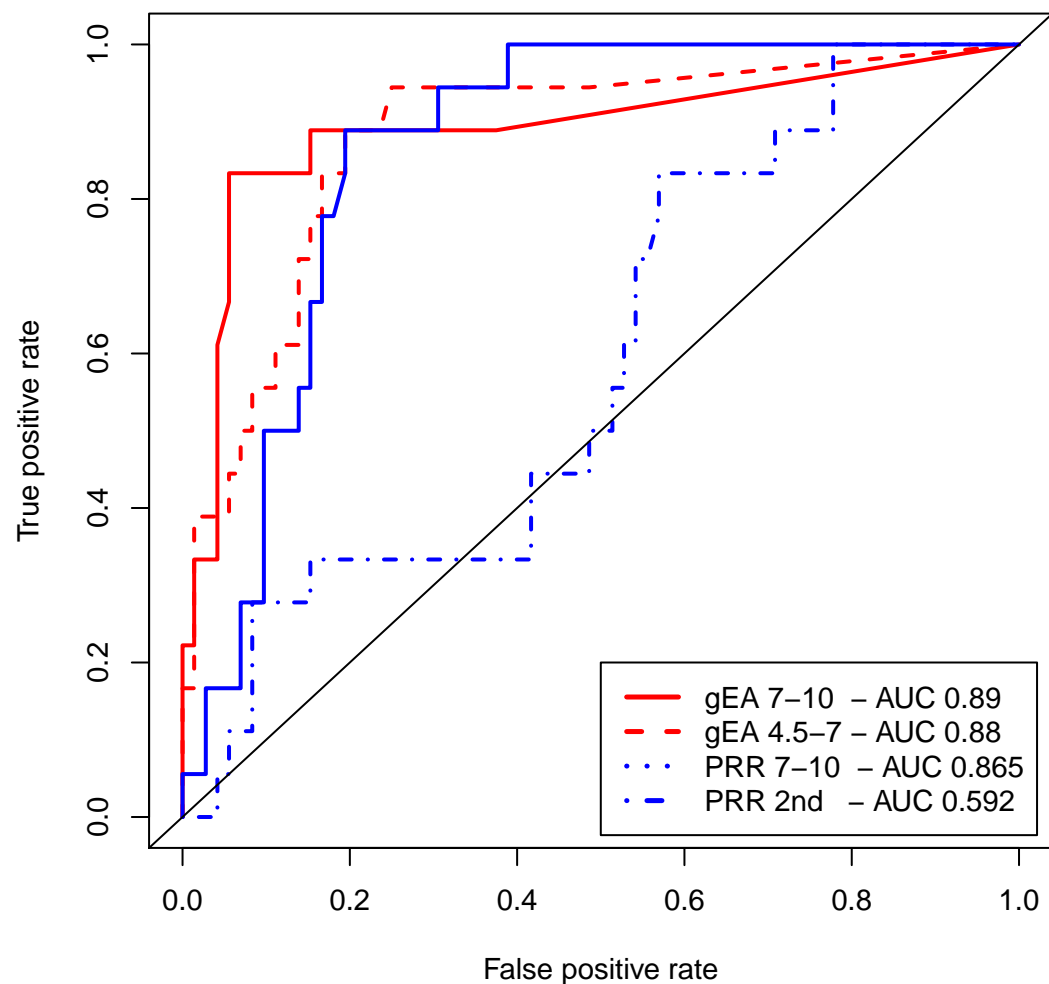

**GI Bleed**

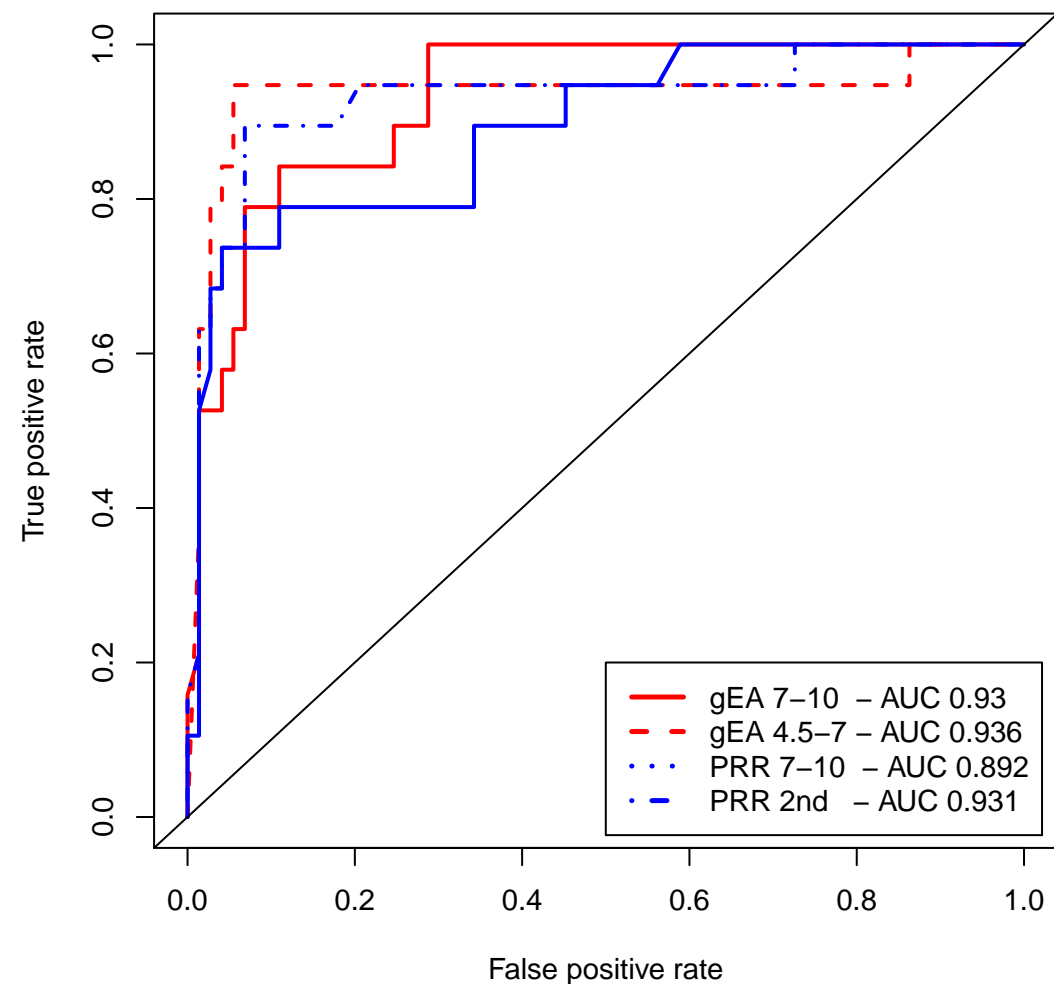

Supplement: Additional file 1: — Performance of selected signal detection methods for drug classes on the OMOP reference set. Performance is measured for each of the four AE outcomes in terms of AUC using all achievable combinations of true positive and false positive rates after grouping the drugs into drug classes (ATC4). The performance of the different approaches is similar for each of the four outcomes, with the one exception of PRR 2nd level on acute myocardial infarction (AUC .612). Overall, both GEA and PRR methods perform best on GI bleed (AUCs from .897 to .935) and poorest on acute liver injury (AUCs from .812 to .853). Using PRR and AE terms aggregated to terms at IC 7–10 improved performance in detecting associations with drug classes (ATC4) only for the outcomes GI bleed and acute myocardial infarction. (PDF 9 kb) [file 12859_2016_1080_MOESM1_ESM.pdf]

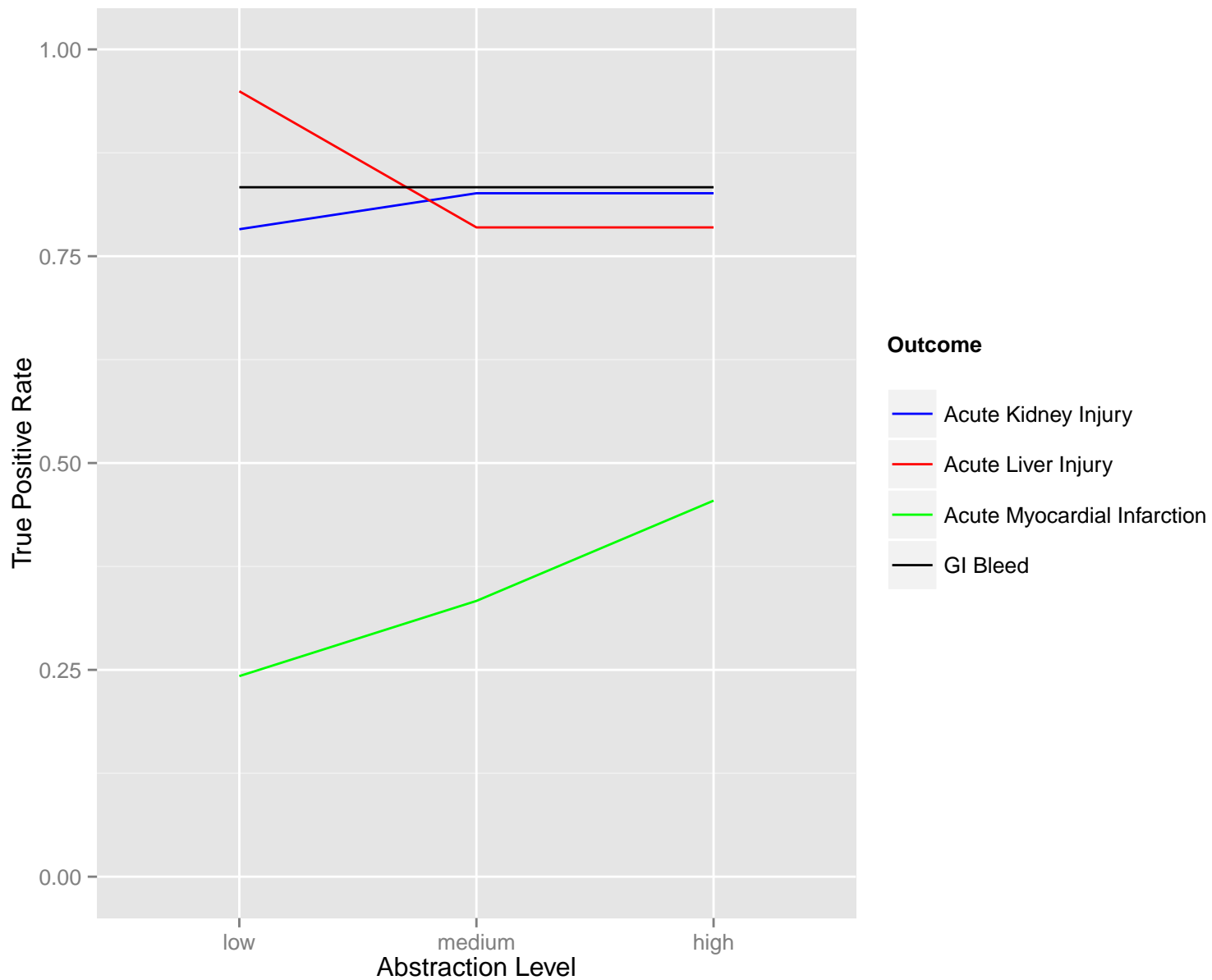

Supplement: Additional file 5: — True Positive Rates for the four OMOP outcomes at different abstraction levels. Drugs that are positively associated to “Acute Myocardial Infarction” in OMOP tend to be annotated in the literature (if at all) with more high-level terms, such as “Heart Diseases” or “Cardiovascular Diseases”. Thus, the number of true positive findings with GEA at a high abstraction level (IC 1–5.5) is double that from the number at low abstraction level (IC 7–10) for AMI. (PDF 4 kb) [file 12859_2016_1080_MOESM5_ESM.pdf]
